# Reproducibility of Rolandic beta rhythm modulation in MEG and EEG

**DOI:** 10.1152/jn.00267.2021

**Published:** 2022-01-19

**Authors:** Mia Illman, Kristina Laaksonen, Veikko Jousmäki, Nina Forss, Harri Piitulainen

**Affiliations:** ^1^Faculty of Sport and Health Sciences, University of Jyväskylä, Jyvaskylä, Finland; ^2^Department of Neuroscience and Biomedical Engineering, Aalto University School of Science, Espoo, Finland; ^3^Aalto NeuroImaging, Aalto University School of Science, Espoo, Finland; ^4^Department of Neurology, Helsinki University Hospital and Clinical Neurosciences, Neurology, University of Helsinki, Helsinki, Finland

**Keywords:** cortical oscillation, cutaneous stimulus, event-related desynchronization, event-related synchronization, passive movement

## Abstract

The Rolandic beta rhythm, at ∼20 Hz, is generated in the somatosensory and motor cortices and is modulated by motor activity and sensory stimuli, causing a short lasting suppression that is followed by a rebound of the beta rhythm. The rebound reflects inhibitory changes in the primary sensorimotor (SMI) cortex, and thus it has been used as a biomarker to follow the recovery of patients with acute stroke. The longitudinal stability of beta rhythm modulation is a prerequisite for its use in long-term follow-ups. We quantified the reproducibility of beta rhythm modulation in healthy subjects in a 1-year-longitudinal study both for MEG and EEG at *T*_0_, 1 month (*T*_1-month_, *n* = 8) and 1 year (*T*_1-year_, *n* = 19). The beta rhythm (13–25 Hz) was modulated by fixed tactile and proprioceptive stimulations of the index fingers. The relative peak strengths of beta suppression and rebound did not differ significantly between the sessions, and intersession reproducibility was good or excellent according to intraclass correlation-coefficient values (0.70–0.96) both in MEG and EEG. Our results indicate that the beta rhythm modulation to tactile and proprioceptive stimulation is well reproducible within 1 year. These results support the use of beta modulation as a biomarker in long-term follow-up studies, e.g., to quantify the functional state of the SMI cortex during rehabilitation and drug interventions in various neurological impairments.

**NEW & NOTEWORTHY** The present study demonstrates that beta rhythm modulation is highly reproducible in a group of healthy subjects within a year. Hence, it can be reliably used as a biomarker in longitudinal follow-up studies in different neurological patient groups to reflect changes in the functional state of the sensorimotor cortex.

## Graphical abstract

**Figure:**
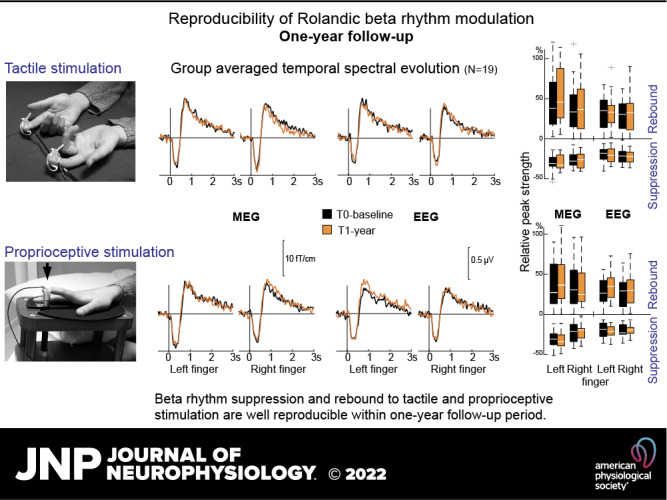

## INTRODUCTION

Oscillatory activity in the sensorimotor cortex at rest is dominated by the ∼20-Hz beta rhythm, which attenuates as a result of the person’s voluntary movement (1), evoked passive movement, or imagined movement (2–6). In addition, the ∼20-Hz beta rhythm is modulated by somatosensory afferent stimuli, such as tactile or electrical stimulation (7–11). The beta rhythm is suppressed briefly after the onset of a stimulus or before self-paced movement. This so-called beta suppression (or event-related desynchronization; ERD) is thought to reflect the excitation of the sensorimotor cortex (12, 13). The suppression is followed by an increase of the beta rhythm above baseline level. This beta rebound (or event-related synchronization; ERS) is associated with neural deactivation or inhibition of the sensorimotor cortex (3, 14, 15). The generator area of the rebound is usually located more anterior than the suppression in the sensorimotor cortex (7, 16, 17). The rebound and suppression are regulated by distinct subunits of GABAergic interneurons (18–21).

Alterations in beta suppression and rebound have been reported in various neurological and psychiatric patient groups, such as stroke (22, 23), schizophrenia (24, 25), Parkinson’s disease (26–28), and cerebral palsy (29–31). Longitudinal studies in patients with stroke have revealed that the strength of the sensorimotor cortex beta rebound correlates with recovery of motor function after acute stroke (11, 32, 33). Consequently, the beta rhythm modulation has been considered as a biomarker of the inhibitory state of the sensorimotor cortex, and it may thus be useful in the evaluation of changes in cortical inhibition during development, aging, and various interventions and the recovery process after brain injury, such as stroke. Espenhahn et al. (34) found the beta rhythm modulation to be well reproducible within a few weeks, but no previous study has investigated the reproducibility of beta suppression and rebound in longer-term measurements, to prove its feasibility for follow-up studies.

The primary aim of the present study was to examine the reproducibility of beta rhythm modulation to tactile and proprioceptive stimulation over a period of 1 year in healthy individuals separately for magnetoencephalography (MEG) and electroencephalography (EEG). In addition, reproducibility of baseline beta power was assessed, as it may affect the estimation of the relative suppression and rebound strengths. Based on previous experiments, indicating a high or excellent reproducibility of MEG and EEG measures related to somatosensory stimuli (35, 36), we hypothesized that the beta rhythm modulation is a reproducible measure when using both MEG and EEG. Stability of the beta modulation over a long period is necessary for its reliable use in clinical follow-up studies.

## MATERIALS AND METHODS

### Subjects

Twenty-one healthy subjects in total were recruited for the study. Nineteen of them (10 females, age 19–35, means ± SD: 23 ± 5 year) were able to complete the 1-year follow-up (13 ± 1.3 month). Additional 1-month follow-up recordings (31 ± 2 days) were performed for 8 (4 females, age 19–31, means ± SD: 25 ± 4 year) of the 21 subjects. All the subjects were right-handed (85 ± 12 on the scale from −100 to 100) according to Edinburgh Handedness Inventory score (37), and had no medication affecting their central nervous system (CNS).

The Aalto University Research Ethics Committee approved the study in accordance with the Declaration of Helsinki. The subjects were asked to sign written informed consent before all follow-up measurements.

### Experimental Design

Reproducibility of the sensorimotor cortex beta rhythm suppression and rebound was assessed between baseline *T*_0_ and 1-year *T*_1-year_ follow-up (*n* = 19) and between baseline *T*_0_ and a 1-month *T*_1-month_ (*n* = 8) measurement sessions.

**Figure 1.**
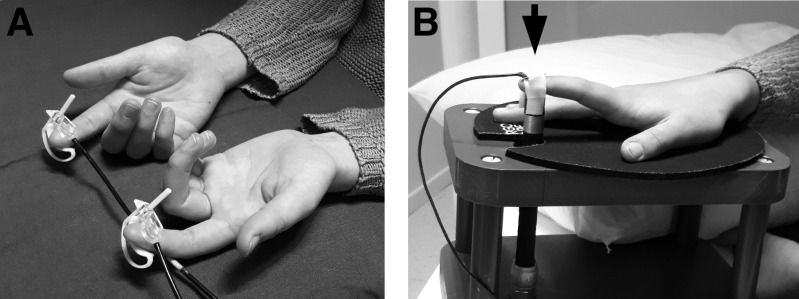
The experimental setup for magnetoencephalography (MEG) compatible tactile (*A*) and proprioceptive (*B*) stimulators.

During the combined MEG/EEG measurement, the subject was fixating at a picture in front of them (size 12 × 15 cm, distance of 2.2 m), while the index fingers were stimulated with tactile and proprioceptive stimuli (Fig. 1) in two separate recordings, respectively. The order of the recordings was randomized. Stimulus-related potential auditory and visual contamination were prevented by using earplugs and visual barrier, respectively. The subject was asked not to pay attention to the stimuli. The total duration of measurement in the magnetically shielded room (MSR) was ∼45 min, and the tactile and proprioceptive stimulus periods lasted ∼9 min each.

#### Tactile stimulation.

Tactile stimuli were given alternately to the left and right hand index fingers every 3 s. The stimuli were produced with Aalto NeuroImaging in-house built pneumatic stimulator utilizing pneumatic diaphragms (4-D NeuroImaging Inc., San Diego, CA) driven by compressed air (4 bar) with a stimulus duration of 180 ms, peaking at 40 ms. The subject held their hands relaxed on a pillow during the stimulation.

#### Proprioceptive stimulation.

The proprioceptive stimuli were evoked to the left and right index finger in separate recordings. A mechanical movement actuator system (38), built at Aalto University, was used to evoke fast flexion-extension movement of the index finger every 5 s (duration 130 ms, mechanical delay from the trigger pulse 35 ms). The movement kinematics were recorded with an MEG-compatible three-axis accelerometer system, built at Aalto NeuroImaging based on an ADXL335 iMEMS Accelometer (Analog Devices Inc., Norwood, MA) attached on the index finger. Compressed air (4 bar) was applied to the actuator resulting in a movement range of ∼5 mm. To minimize possible tactile sensation of the fingertip, the index finger was taped with surgical tape. To confirm the correct finger position during the measurement, the finger was lightly taped to the actuator and the stimulated hand was supported in a comfortable relaxed position with pillows.

### Data Acquisition

The simultaneous MEG/EEG measurements were recorded at Aalto University (MEG Core, Aalto NeuroImaging), with a 306-channel (204 planar gradiometers, 102 magnetometers) whole scalp MEG system (Elekta Neuromag, Elekta Oy, Helsinki, Finland). A 60-channel MEG-compatible EEG cap (ANT Neuro waveguard original) with Ag-AgCl surface electrodes mounted according to the international 10-20 system, was used for EEG recordings. In addition, eye blink artifacts were detected with two vertical electro-oculogram electrodes (EOG). The MEG/EEG recordings were performed in a magnetically shielded room (MSR; Imedco AG, Hägendorf, Switzerland). Before the recordings, two indicator coils were attached above the ears and three onto the forehead of the EEG cap. The location of these five coils, three anatomical landmarks (left and right preauricular points and nasion) and additional 100–200 points from the surface of head, were determined with a three-dimensional (3-D) digitizer (Fastrak 3SF0002, Polhemus Navigator Sciences, Colchester, VT). The head position was determined in the beginning of each measurement session and continuously during the measurement by sending a low current to the indicator coils and detecting the position of the coils with respect to the MEG sensor array.

A sampling frequency of 1000 Hz and bandpass filter 0.1–330 Hz was used in MEG, EEG, and accelerometer recordings. The impedances of the EEG electrodes were verified to be below 5–10 kΩ before the recording.

### Data Processing and Analysis

#### Preprocessing.

A custom-made MATLAB script was used to transform the MEG raw signals from the different measurement sessions (*T*_0_, *T*_1-month_, *T*_1-year_) to the same average head-coordinate system, separately to tactile and proprioceptive stimuli, for each subject. This improves the comparability of different measurement sessions when the obtained reference head positions are used for coordinate matching in the Maxfilter software (v2.2; Elekta Oy, Helsinki, Finland). MEG raw signals were filtered with the signal-space separation method with temporal extension (tSSS) and head movement compensation (threshold 25 mm) was obtained (39). The following parameters were used in the Maxfilter software: buffer length 16 s, subspace correlation limit 0.98, inside expansion order 8, and outside expansion 3.

Hereafter, the MEG and EEG data were analyzed with MNE Python (v. 0.17) (40). The EEG signals were re-referenced to the average reference over all good quality channels, individually for each subject. Eye blink artifacts (two magnetometer and two gradiometer components) were removed with principal component analysis (PCA; 41). Evoked responses related to stimulus onset, which can disturb the baseline detection of the beta modulation, were subtracted from each epoch from both MEG and EEG data (42).

#### Determination of beta rhythm modulation.

The temporal spectral evolution (TSE) method was used to quantify the strength of the stimulus-related beta rhythm modulation in the follow-up measurements (7). MEG and EEG data were first filtered to a 13- to 25-Hz frequency band (a symmetric linear-phase FIR filter with a transition band of 1 Hz at the low- and high cutoff frequency and Hamming window, filter length 3.3), which in a previous study has been found to show the strongest beta rhythm modulation for all subjects (43). The lower beta frequencies are needed specifically to detect the beta rebound (5, 29, 44). After band-pass filtering, a Hilbert transform was applied to obtain the envelope signal, after which the data were averaged from –500 to 3,000 ms with respect to the stimulus trial. Peak strengths and latencies of the beta rhythm suppression and rebound were determined from the individual TSE curves. MEG and EEG channels used for rebound/suppression determination were individually selected over the sensorimotor cortex areas and they remained the same (within one subject) in all sessions. Channels were selected based on the strongest response, noticing that in some subjects the suppression and rebound were more pronounced in different channels (one or two channels in one hemisphere). The baseline beta rhythm power was determined from these individually selected MEG and EEG channels from a time window of −500 to −100 ms, and the absolute suppression and rebound strengths were converted to relative values (in percentage) with respect to the prestimulus baseline from –500 to –100 ms to allow better comparability between different subjects and measurement sessions. The interstimulus intervals of the stimuli were chosen to allow a return of the beta rhythm to baseline level well before next stimulus onset, i.e., to keep the baseline stable during the measurement.

Beta rhythm modulation to tactile and proprioceptive stimuli was visualized with topographic TSE maps and time-frequency representations (TFRs; 45) averaged over all subjects in both MEG and EEG. TFRs, in the frequency range of 3–36 Hz and a time window of −700 to 3,200 ms with respect to stimulus onset, were calculated using Morlet wavelets by scaling the number of cycles by frequency (*f*/2).

### Statistical Analysis

Statistical tests were performed with IBM SPSS Statistics (v. 27.0. Armonk, NY, IBM Corp). The Shapiro–Wilk test was used to test the normality of the data. The latencies and relative peak strengths of the beta rhythm suppression and rebound turned out to be not normally distributed, and therefore the nonparametric Wilcoxon test was used to test differences in the latency and strength of beta suppression and rebound between the follow-up measurements.

Correlations of beta suppression and rebound strengths between the follow-up measurements were determined with Spearman’s correlation coefficient test. The reproducibility of suppression and rebound was in addition tested with the intraclass correlation coefficient (ICC) with two-way random effects and absolute agreement. In addition, coefficient of variation (CV) was defined to show interindividual variability of beta suppression and rebound at *T*_0_, *T*_1-month_, and *T*_1-year._

The effect of multiple tests was corrected with Bonferroni correction. A *P* value between 0.05 and 0.001 was used to assess significance.

## RESULTS

A consistent number of trials (means ± SD) were collected for the TSE analysis between *T*_0_ and *T*_1-month_ follow-up measurements to tactile (105 ± 11 vs. 101 ± 6) and proprioceptive (108 ± 12 vs. 101 ± 10) stimulation. As can be seen from the results, the number of trials was higher at *T*_0_ than at *T*_1-year_ measurements to tactile (105 ± 11 vs. 92 ± 13, *P* > 0.001) and proprioceptive (108 ± 12 vs. 99 ± 7, *P* > 0.001) stimulation.

### Spatiotemporal Characteristics of Beta Rhythm Modulation

#### Spatial distribution of beta suppression and rebound.

**Figure 2.**
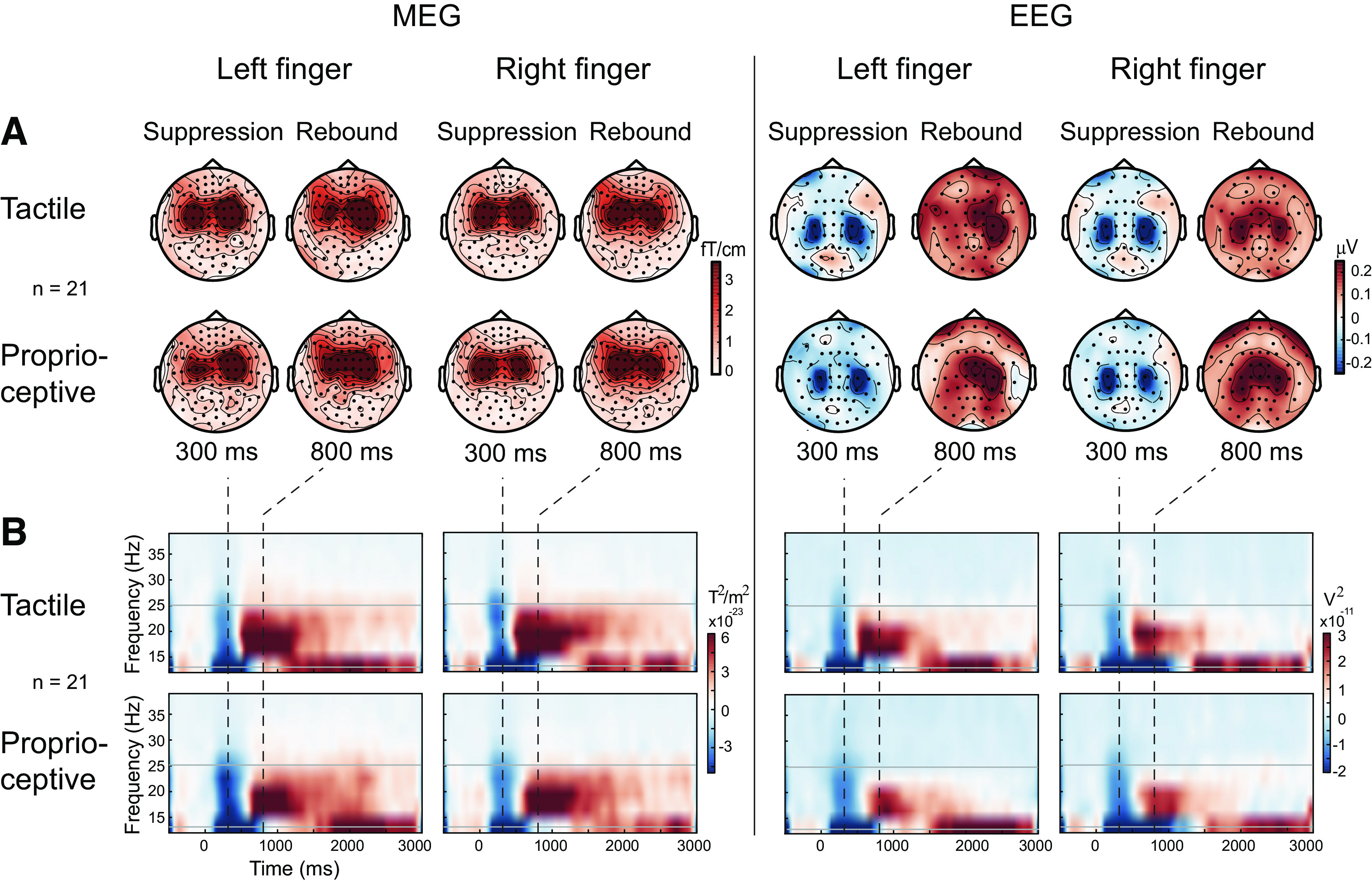
Grand averaged (*n* = 21 subjects) topographic distributions and time frequency representations (TFR) of the beta rhythm modulation to tactile and proprioceptive stimulation in the baseline *T*_0_ measurement. *A*: topographic maps show magnetic field strengths (magnetoencephalography, MEG) and electrical scalp potentials (electroencephalography, EEG) of the beta suppression and rebound to left and right stimuli. Note that MEG topographies reflect the vector sum of the gradiometer pairs, and thus obtain only positive values. *B*: TFR images illustrates temporal evolution of the beta frequency power from one of the most representative gradiometer over the sensorimotor cortex contralateral to the stimulation with respect to trigger onset at 0 s. Black dashed lines indicate the time instants if the suppression and rebound illustrated in *A*. Gray lines indicate the beta frequency band used in temporal spectral evolution (TSE) analysis.

Figure 2*A* illustrates group averaged (*n* = 21) spatial distribution of beta rhythm suppression and rebound at *T*_0_ both in MEG and EEG. Beta suppression and rebound were observed bilaterally over the sensorimotor cortex shortly after the onset of both tactile and proprioceptive stimuli, with stronger responses in the contralateral hemisphere (especially rebound) in relation to the stimulated hand. These contralateral responses were taken for further analysis.

#### Time-frequency representation.

Figure 2*B* shows contralateral beta rhythm modulations (group averaged over 21 subjects) to tactile and proprioceptive stimuli at *T*_0_. The decrease of beta rhythm is most pronounced at 250–350 ms and subsequently increased at 700–850 ms after the onset of tactile and proprioceptive stimuli.

### Reproducibility of Beta Suppression and Rebound

#### Reproducibility within 1 year.

##### Latencies.

Mean latencies of beta suppression and rebound for both stimuli in MEG and EEG are shown in Table 1. No statistically significant differences (*P* > 0.28) in suppression or rebound latencies were observed between the different measurements (*T*_0_, *T*_1-month_, and *T*_1-year_) and stimuli.

**Table 1.** Relative peak strengths and latencies of the beta rhythm suppression and rebound in three follow-up MEG/EEG measurements

	Tactile Stimulation				Proprioceptive Stimulation			
	MEG		EEG		MEG		EEG	
	LH	RH	LH	RH	LH	RH	LH	RH
			*Suppression*					
*T*_0_								
Relative amplitude, %	−29 ± 2	−25 ± 2	−19 ± 2	−19 ± 2	−31 ± 2	−23 ± 3	−20 ± 2	−20 ± 2
SD ±	10	10	9	10	11	12	9	8
CV, %	34	40	47	47	35	52	45	40
Peak latency, ms	260 ± 17	296 ± 17	247 ± 22	263 ± 17	320 ± 22	316 ± 20	304 ± 27	299 ± 17
*T*_1-month_								
Relative amplitude, %	−28 ± 4	−23 ± 5	−21 ± 3	−15 ± 4	−30 ± 4	−23 ± 5	−23 ± 4	−22 ± 3
SD ±	12	14	9	10	12	14	12	10
CV, %	42	61	45	67	40	61	52	45
Peak latency, ms	213 ± 24	250 ± 38	224 ± 36	248 ± 39	232 ± 29	247 ± 29	339 ± 37	250 ± 26
*T*_1-year_								
Relative amplitude, %	−30 ± 2	−27 ± 2	−20 ± 2	−23 ± 2	−33 ± 2	−21 ± 3	−22 ± 2	−20 ± 2
SD ±	9	10	9	7	10	13	7	8
CV, %	30	37	45	30	30	62	32	40
Peak latency, ms	255 ± 22	255 ± 15	291 ± 21	250 ± 21	341 ± 24	311 ± 19	361 ± 18	281 ± 22
			*Rebound*					
*T*_0_								
Relative amplitude, %	47 ± 8	37 ± 6	34 ± 4	30 ± 4	41 ± 7	36 ± 6	29 ± 4	27 ± 4
SD ±	35	29	20	19	31	28	17	17
CV, %	74	78	59	63	76	78	59	63
Peak latency, ms	729 ± 38	785 ± 57	703 ± 38	750 ± 47	893 ± 56	891 ± 58	845 ± 42	792 ± 37
*T*_1-month_								
Relative amplitude, %	59 ± 16	50 ± 17	45 ± 9	46 ± 8	53 ± 10	53 ± 14	41 ± 7	35 ± 8
SD ±	45	48	24	22	30	39	19	23
CV, %	76	96	53	48	57	74	46	66
Peak latency, ms	765 ± 47	690 ± 85	724 ± 81	618 ± 66	866 ± 97	855 ± 91	813 ± 71	739 ± 60
*T*_1-year_								
Relative amplitude, %	54 ± 8	40 ± 8	34 ± 5	33 ± 5	43 ± 7	37 ± 6	35 ± 4	30 ± 4
SD ±	35	34	20	24	32	27	17	18
CV, %	65	85	59	73	74	73	49	60
Peak latency, ms	711 ± 38	854 ± 82	722 ± 43	719 ± 57	889 ± 47	900 ± 68	897 ± 64	849 ± 46

Values (mean ± SE) are presented for contralateral responses to stimulated hand (LH, left hand; RH, right hand) for both tactile and proprioceptive stimulation. In addition, standard deviation (SD) and coefficient of variation (CV) are shown for the suppression and rebound strengths. The number of subjects is *n* (*T*_0_) = 21, *n* (*T*_1-month_) = 8, *n* (*T*_1-year_) = 19. EEG, electroencephalography; MEG, magnetoencephalography; *T*_0_, baseline; *T*_1-month_, follow-up after 1 month; *T*_1-year_, follow-up after 1 year.

##### Strength of beta suppression and rebound.

**Figure 3.**
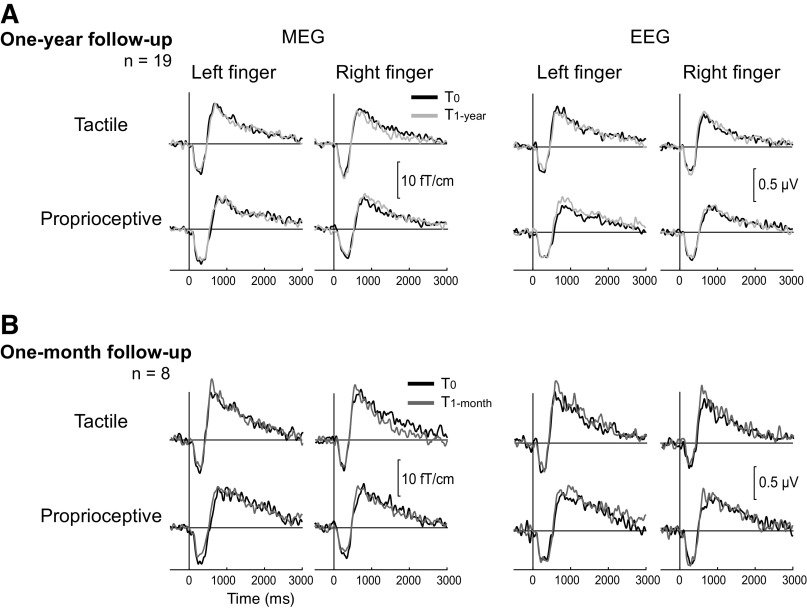
Grand averaged beta rhythm modulation to tactile and proprioceptive stimuli in the baseline and follow-up measurements. One-year (*T*_1-year_, *n* = 19) (*A*) and 1-month (*T*_1-month_, *n* = 8) (*B*) follow-up measurements are compared with the baseline (*T*_0_) measurement, not showing significant differences between the measurements. Temporal spectral evolution (TSE) curves are showing the peak modulation of the most representative magnetoencephalography (MEG) and electroencephalography (EEG) channels over the sensorimotor cortex contralateral to the stimulated hand. Trigger onsets are shown as vertical lines at zero time; *n*, Number of subjects.

Figure 3*A* shows group averaged (*n* = 19) TSE curves to tactile and proprioceptive stimuli at *T*_0_ and *T*_1-year_. Beta rhythm suppression and rebound are well identifiable in all sessions both in MEG and EEG, and the suppression and rebound strengths appear similar between *T*_0_ and *T*_1-year_ sessions. Supplemental Fig. S1 (see https://doi.org/10.6084/m9.figshare.17032178.v1) shows the individual TSE curves for all subjects at three different measurement sessions.

**Figure 4.**
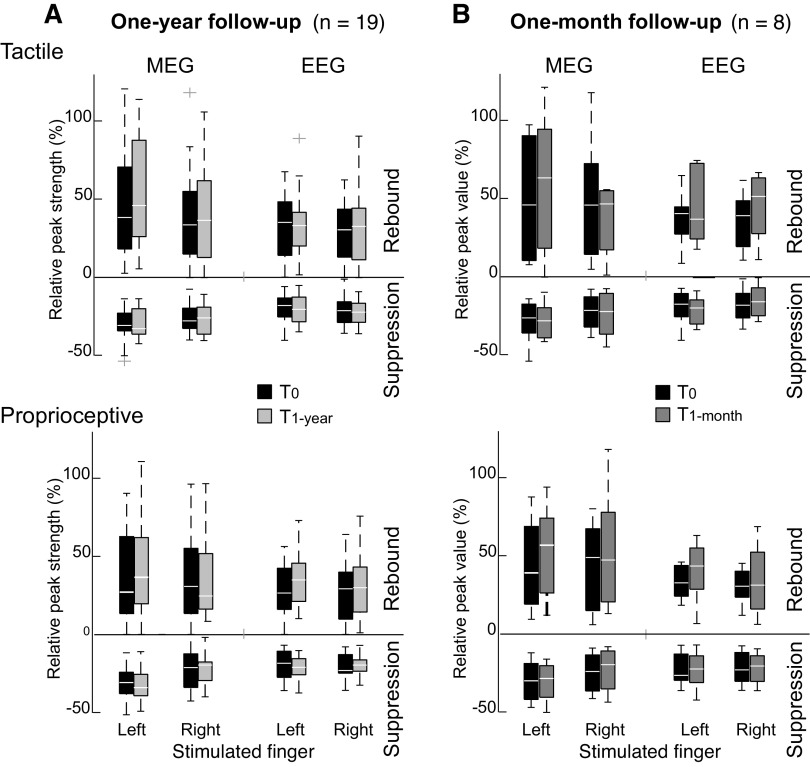
Peak strength of beta rhythm suppression and rebound to tactile and proprioceptive stimuli relative to baseline value for 1-year (*A*) and 1-month (*B*) follow-up measurement. Fifty percent of strength values are included in the box, horizontal lines indicate median value, and whiskers indicate variability outside the upper and lower quartiles. Outlier values are shown as crosses. EEG, electroencephalography; MEG, magnetoencephalography; *n*, number of subjects.

Figure 4*A* illustrates the relative peak strengths (% to baseline) of beta suppression and rebound at *T*_0_ and *T*_1-year_ both in MEG and EEG to left and right finger stimulation. Beta suppression and rebound strengths did not differ significantly (MEG *P* = 1.0; EEG *P* > 0.053) between the 1-year follow-up measurements (*T*_0_ vs. *T*_1-year_, *n* = 19). Mean values and standard deviations of the relative peak strengths for beta suppression and rebound are shown in Table 1.

##### Intersession correlations.

**Figure 5.**
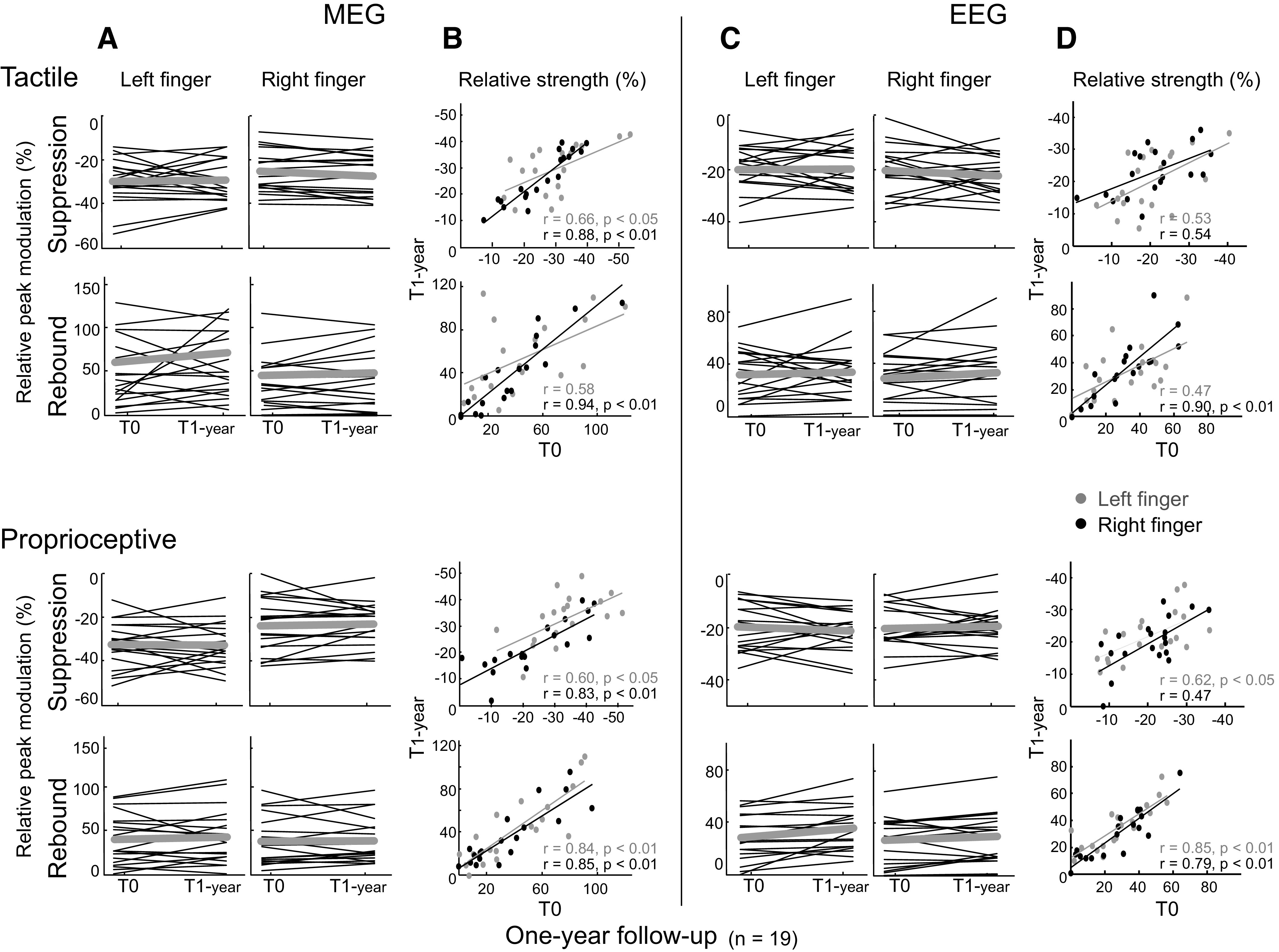
Individual subjects’ beta suppression and rebound strengths in the baseline (*T*_0_) and 1-year follow-up measurements (*n* = 19) to tactile and proprioceptive stimulations in magnetoencephalography (MEG) and electroencephalography (EEG). Relative peak modulations for each subject in the baseline (*T*_0_) and 1-year follow-up (*T*_1-year_) measurements for left and right hand stimuli for MEG (*A*) and EEG (*C*). Thin black lines represent direction of change for each subject separately, and gray lines the group-mean changes. Scatterplots and Spearman’s correlation coefficients for the relative peak modulation strengths between the *T*_0_ and *T*_1-year_ measurements for MEG (*B*) and EEG (*D*). The gray color represents left and black color right hand stimulation.

Figure 5*A* presents the relative peak strengths of beta suppression and rebound individually (*n* = 19) at *T*_0_ and *T*_1-year_. The suppression and rebound strengths are well reproducible both in MEG and EEG for most of the subjects. Intraclass correlation coefficient values indicated good to excellent intersession reproducibility for suppression 0.72–0.96 and rebound 0.70–0.95 strengths. However, the ICC values appeared to be stronger for the dominant compared with the nondominant hand. Figure 5*B* shows scatterplots respectively for suppression and rebound strengths between *T*_0_ and *T*_1-year_ measurements. The beta suppression and rebound strengths to tactile and proprioceptive stimuli correlated significantly between the measurements; the Spearman’s correlation coefficients (*r*) for the suppression and rebound are 0.47–0.88 and 0.47–0.94, respectively. More detailed correlation values are shown in Table 2.

**Table 2.** Intersession correlations of the beta rhythm suppression and rebound relative strengths for both tactile and proprioceptive stimulation in MEG and EEG

	MEG				EEG			
	Left Hand		Right Hand		Left Hand		Right Hand	
	ICC	*r*	ICC	*r*	ICC	*r*	ICC	*r*
Tactile stimulus								
Suppression								
*T*_0_ vs. *T*_1-year_ (*n* = 19)	0.75	0.66*	0.96	0.88**	0.73	0.53	0.72	0.54
*T*_0_ vs. *T*_1-month_ (*n* = 8)	0.84	0.74	0.96	0.91*	0.87	0.71	0.46	0.50
Rebound								
*T*_0_ vs. *T*_1-year_ (*n* = 19)	0.70	0.58	0.95	0.94**	0.75	0.47	0.90	0.90**
*T*_0_ vs. *T*_1-month_ (*n* = 8)	0.91	0.74	0.95	0.91*	0.74	0.71	0.82	0.83
Proprioceptive stimulus								
Suppression								
*T*_0_ vs. *T*_1-year_ (*n* = 19)	0.76	0.60*	0.88	0.83**	0.76	0.62*	0.80	0.47
*T*_0_ vs. *T*_1-month_ (*n* = 8)	0.88	0.79	0.96	0.86*	0.79	0.76	0.87	0.74
Rebound								
*T*_0_ vs. *T*_1-year_ (*n* = 19)	0.92	0.84**	0.93	0.85**	0.87	0.85**	0.93	0.79**
*T*_0_ vs. *T*_1-month_ (*n* = 8)	0.90	0.81	0.93	0.95**	0.75	0.60	0.83	0.95**

Intraclass (ICC) and Spearman’s (*r*) correlation coefficient values are presented for contralateral responses to stimulated hand. EEG, electroencephalography; MEG, magnetoencephalography; *T*_0_, baseline; *T*_1-month_, follow-up after 1 month; *T*_1-year_, follow-up after 1 year.

* *P* < 0.05;

** *P* < 0.01.

In summary, the strength of beta rhythm suppression and rebound to tactile and proprioceptive stimuli both in MEG and EEG were highly reproducible in the 1-year follow-up period.

#### Reproducibility within 1 month.

##### Strength of beta suppression and rebound.

The additional 1-month follow-up recordings were performed for a subgroup of our participants to confirm that the reliability of beta rhythm modulation was similar for both the 1-month and 1-year follow up. Figure 3*B* shows group averaged (*n* = 8) TSEs in the *T*_0_ and *T*_1-month_ measurements. The relative peak strengths of suppression and rebound (seen in Table 1) did not differ significantly (*P* = 1.0) between the *T*_0_ and *T*_1-month_ measurements.

##### Intersession correlations.

The beta suppression and rebound relative peak strengths between *T*_0_ and *T*_1-month_ measurements correlated strongly in MEG, but correlations were weaker in EEG. The ICC and Spearman’s correlation coefficient values between *T*_0_ and *T*_1-month_ measurements are shown in Table 2.

### Reproducibility of Baseline Beta Power

Baseline beta rhythm power, and Spearman’s correlation and ICC coefficients for tactile and proprioceptive stimulation in MEG and EEG are shown in Table 3. Baseline beta power between *T*_0_ and *T*_1-month_ or *T*_0_ and *T*_1-year_ measurements did not show significant differences.

**Table 3.** Baseline beta power (means ± SE) and intraclass (ICC) and Spearman’s (r) correlation coefficient values on the sensorimotor cortex in three follow-up MEG/EEG measurements for contralateral responses to stimulated hand

Tactile Stimulation					
MEG, fT/cm	Left hand	Right hand	EEG, µV	Left hand	Right hand
*T*_0_	36.2 ± 3	42.0 ± 4		2.3 ± 0.2	2.4 ± 0.2
*T*_1-month_	33.3 ± 3	41.8 ± 5		2.4 ± 0.4	2.3 ± 0.4
*T*_1-year_	35.5 ± 4	43.2 ± 4		2.3 ± 0.2	2.3 ± 0.2
ICC					
*T*_0_ vs. *T*_1-year_	0.87	0.81		0.95	0.91
*T*_0_ vs. *T*_1-month_	0.84	0.86		0.95	0.96
Spearman’s (*r*)					
*T*_0_ vs. *T*_1-year_	0.80**	0.75**		0.96**	0.88**
*T*_0_ vs. *T*_1-month_	0.81*	0.91**		0.98**	0.99**

Proprioceptive Stimulation					
MEG, fT/cm	Left hand	Right hand	EEG, µV	Left hand	Right hand
*T*_0_	32.4 ± 3	39.1 ± 4		2.4 ± 0.2	2.3 ± 0.2
*T*_1-month_	31.0 ± 4	37.6 ± 5		2.4 ± 0.5	2.6 ± 0.5
*T*_1-year_	33.2 ± 4	41.1 ± 4		2.4 ± 0.2	2.3 ± 0.2
ICC					
*T*_0_ vs. *T*_1-year_	0.72	0.91		0.94	0.90
*T*_0_ vs. *T*_1-month_	0.76	0.92		0.99	0.95
Spearman’s (*r*)					
*T*_0_ vs. *T*_1-year_	0.57*	0.85**		0.93**	0.87**
*T*_0_ vs. *T*_1-month_	0.57	0.79*		0.96**	0.83*

EEG, electroencephalography; MEG, magnetoencephalography.

* *P* < 0.05;

** *P* < 0.01.

ICC coefficients were good or excellent between *T*_0_ and *T*_1-month_ (0.76–0.99) and *T*_0_ and *T*_1-year_ (0.72–0.95) measurements, and corresponding Spearman’s correlations coefficients were 0.57–0.99 (*T*_0_ vs. *T*_1-month_) and 0.57–0.96 (*T*_0_ vs. *T*_1-year_).

### Interindividual Variation of Beta Suppression and Rebound

Interindividual variation (coefficient of variation) for the relative strength of beta suppression was 30%–67% and for rebound was 46%–96% at *T*_0_, *T*_1-month_, and *T*_1-year_. The coefficient of variation (in %) are shown in Table 1.

## DISCUSSION

These novel results indicate that the beta rhythm modulation, i.e., suppression and rebound are highly reproducible over a long 1-year follow-up period. This information is essential for the usability of these biomarkers in longitudinal follow-up experiments. In addition, the absolute baseline beta power remained at stable level throughout the follow-up period. We used fixed and well repetitive tactile and proprioceptive stimuli to modulate the beta rhythm. Hence, the effects of instabilities, typical for active volitional movements, were eliminated and did not affect the assessment of reproducibility. Our study proves that the reproducibility of beta suppression and rebound within 1 year is good or excellent both when using MEG or EEG, and therefore, the beta rebound can be reliably used as a biomarker to reflect the functional state of the sensorimotor cortex in follow-up studies.

### Reproducibility of Beta Rhythm Modulation

In the current study, the reproducibility of beta suppression and rebound were verified to be good or excellent. Previous studies have reported that the beta rhythm modulation to active movement to be well reproducible within days or weeks in EEG (34, 46).

#### Beta suppression versus rebound.

The beta suppression is mainly thought to reflect the excitation of the SMI cortex to sensory input, whereas the rebound appears later, lasts longer, and is regulated by more complex inhibitory interneuron networks, and is thus more sensitive to alterations in the stimulus or environment. The beta suppression and rebound are generated in slightly different locations in the SMI cortex, with the rebound more anteriorly in the primary motor cortex (MI) (16, 47, 48). The rebound appears to be stronger in the contralateral hemisphere with respect to stimulus, whereas the suppression is similarly strong in both hemispheres (49). Due to these spatiotemporal differences in beta suppression and rebound, they are thought to reflect distinct functional roles in the sensorimotor cortical processing. Consequently, the beta rebound has been shown to be altered in different neurological conditions, such as stroke and schizophrenia, whereas the suppression has shown to remain relatively stable in these conditions and during follow-up (11, 32, 33). It may be that the suppression is more like all-or-nothing type of response, whereas the rebound is more prone to changes in the functional state of the sensorimotor cortex.

#### Active movement versus tactile and proprioceptive stimulation.

Although beta rhythm modulation has been reported to be reproducible for well-controlled active movement (34, 46), active movement-induced beta rebound is susceptible for various factors, such as speed and intensity of movement (48, 50, 51). Movement preparation has been seen to induce the beta rhythm suppression before movement onset (1, 52), and even motor imaging has been shown to cause beta rhythm modulation (4), which can hamper the evaluation of its reproducibility. In patient studies, in particular, slight changes in the performance of the active movement may affect the assessment of the reproducibility of beta modulation and thus interfere in the interpretation of changes in sensorimotor cortex function. Proprioceptive and tactile stimulation are easy to standardize and remain the same throughout the measurement, which is especially important in clinical studies that are otherwise more prone to subject-related disturbances. Taken together, especially in patient studies, tactile or proprioceptive stimulation should preferably be used to study longitudinal changes in sensorimotor cortex function, since it is advisable to keep the measurement settings as stable as possible.

#### 1-month versus 1-year.

The reproducibility of beta modulation has earlier been studied within few weeks (34), and there is no certainty about its reproducibility in longer term. We examined the reproducibility of beta rhythm modulation within 1-year period, to ensure its feasibility for long-term follow-up studies. This is especially important, since the beta rhythm rebound has been proposed to be a biomarker reflecting functional recovery of the SMI cortex after acute stroke, whereas no clear association between suppression and motor recovery has been found (11, 32, 33). The beta power and the strength of beta modulation have been shown to increase in relation to aging (6, 47). However, such changes seem not to occur within a 1-year follow-up period, at least in relatively young adult participants. In older individuals, the aging effect may be more significant, and need to be clarified in future studies. Nevertheless, the present results encourage the use of beta rebound/modulation to evaluate the effectiveness of rehabilitation and drug interventions in short- or long-term follow-up studies. In addition, in well-recovering patients with stroke, the rebound in the affected hemisphere recovered to the level of the unaffected hemisphere within 1 year, although it was diminished in the acute phase and at 1 month (53).

#### Interindividual and intersession variations of beta suppression and rebound.

Beta rhythm suppression and rebound typically show high interindividual variation and are weak and even undetectable in some individuals. The higher interindividual variation likely arises from individual differences in the functional anatomy of the sensorimotor strip. For example, the sensorimotor rhythm generator may be located more on the gyral or fissural cortex affecting the depth and orientation of the strength of the source detected with MEG outside the skull (54). However, the beta suppression has proved to be more stable than the rebound, which is more sensitive to, for example, changes in stimulus properties, such as speed and intensity of movement. In addition, the state of the subject’s alertness may also effect on the strength of beta rhythm modulation. For most of our participants, the beta modulation remained stable at individual level during the 1-year follow-up (on average suppression < 9% and rebound < 26% change), although some participants showed a greater intersession variability (suppression 0.1%–30% and rebound 0%–98%). It is noteworthy to mention that the interindividual variation of beta modulations were ∼30%–62%, but the intersession variation was on average less than <26%. This further indicates that beta modulations are reproducible at group level, but in some individuals the variability can be substantial. Therefore, it is important to standardize the recording design as well as possible, e.g., to pay attention to the homogeneity of the stimuli and the state of the participants alertness during the MEG/EEG registration.

#### MEG versus EEG.

Our study showed high or excellent reproducibility both for MEG and EEG, but ICC values appeared to be higher for MEG than EEG. This is likely to be due to MEG’s better sensitivity to detect beta rhythm modulation. However, the relative suppression and rebound strengths correlated well between MEG and EEG measurements, and therefore both methods are valid for measuring beta modulation (5). Since mainly EEG has been adopted as a standard method in clinical trials, it is important that a neurophysiological biomarker can be reliably and reproducibly detected with it. The present study indicated the feasibility of both MEG- and EEG-based detection of the beta rhythm modulation and utilization in long-term follow-up studies.

### Factors Affecting the Baseline or Induced Beta Power

In healthy individuals, the Rolandic beta power at rest has been shown to be highly reproducible both when assessed with MEG and EEG (34, 55, 56). Typically, the beta suppression and rebound are computed relative (in percentage) to the baseline beta power. For this reason, alterations in baseline beta power during a study may also affect induced beta suppression and rebound strengths (18, 57, 58). There are several factors (major ones are discussed in the following sections) that may alter the baseline level of the beta rhythm power, and hence should be taken into account when using baseline normalized modulation of beta suppression and rebound. However, previous studies have shown that baseline beta power remain the same during stroke recovery (59, 60), although the beta modulation amplitudes show prominent changes during the recovery period (11). In other words, the beta rhythm resting power and induced modulation strength appear to be distinct phenomena likely reflecting different aspects in cortical sensorimotor processing.

#### Age.

The beta rhythm has been shown to be age dependent. In children, the beta power has shown to be reduced than in adults (61). Concomitantly, several studies have shown that in elderly subjects the beta power at rest is increased than in younger subjects, leading to an increase of beta suppression (6, 47, 57, 62, 63), with the exception of Alzheimer’s disease, where the resting-state beta power has been shown to decrease (64). The frequency of the beta rhythm has also been shown to be lower with increasing age (63).

#### Circadian rhythm.

The circadian rhythm is known to affect the level of the beta rhythm power, being lower in the morning and increasing toward the afternoon (46, 65). Also the strength of beta suppression has been shown to increase toward the afternoon, but no such effect has been observed for the beta rebound (46).

#### Drugs.

Drugs that affect the GABAergic neurotransmitter system have been observed to alter the intensity of the beta rhythm. Benzodiazepine, a nonselective GABA_A_ agonist elevates the beta rhythm power and increases the strength of beta suppression (19, 21, 66, 67). In contrast, tiagabine (GABA reuptake transporter, which affects both GABA_A_ and GABA_B_ subunits) has been shown to increase the beta power and amplitude of beta suppression, but decrease the amplitude of beta rebound (18).

#### Alertness and attention.

Mental fatigue caused by long-lasting attentive task and overload has been shown to enhance the beta power (68), whereas reduced alertness, for example, due to sleepiness decreases the beta power and the amplitude of beta suppression and rebound (43). Enhanced vigilance and active attention to stimuli have also been shown to increase the beta power (69, 70), and either to increase (70, 71) or decrease (72) the intensity of beta suppression and rebound. In addition, cortical proprioceptive processing is altered when attention is directed to the proprioceptive stimuli, increasing the sustained-evoked field amplitude but reducing the beta power (73).

In the present study, all these confounding factors were strived to standardize as accurately as possible; measurements were taken at the same time of day, age distribution of the subjects was even, the subjects had no CNS medication, and they were instructed to keep good vigilance and not to pay attention to the stimuli during the recordings.

### Conclusions

Our study demonstrates that the beta rhythm suppression and rebound to tactile and proprioceptive stimulation are reproducible both in MEG and EEG recordings within a 1-year period. This finding suggests that the beta modulation is a suitable tool for longitudinal studies to monitor changes in the level of sensorimotor cortex activation and inhibition. Such a need has arisen, for example, in evaluation of the effectiveness of rehabilitation and drug intervention in neurological patients. Our results encourage a wider use of beta rhythm modulation, especially the beta rebound, as a biomarker to study and follow-up the function of sensorimotor cortex.

## SUPPLEMENTAL DATA

10.6084/m9.figshare.17032178.v1 Supplemental Fig. S1: https://doi.org/10.6084/m9.figshare.17032178.v1.

## GRANTS

This work was supported by the SalWe Research Program for Mind and Body, Tekes—the Finnish Funding Agency for Technology and Innovation under Grant Number 1104/10; Academy of Finland under Grant Numbers 296240, 307250, 326988, 327288; Aalto NeuroImaging, Aalto Brain Center; and Jane and Aatos Erkko Foundation Grant number 602.274.

## DISCLOSURES

No conflicts of interest, financial or otherwise, are declared by the authors.

## AUTHOR CONTRIBUTIONS

M.I., K.L., and H.P. conceived and designed research; M.I. performed experiments; M.I. analyzed data; M.I., H.P., and K.L. interpreted results of experiments; M.I. prepared figures; M.I. drafted manuscript; M.I., K.L., V.J., N.F., and H.P. edited and revised manuscript; M.I., K.L., V.J., N.F., and H.P. approved final version of manuscript.

## References

[1] Pfurtscheller G, Lopes da Silva FH. Event-related EEG/MEG synchronization and desynchronization: basic principles. Clin Neurophysiol 110: 1842–1857, 1999. doi:10.1016/s1388-2457(99)00141-8. 10576479

[2] Alegre M, Labarga A, Gurtubay IG, Iriarte J, Malanda A, Artieda J. Beta electroencephalograph changes during passive movements: sensory afferences contribute to β event-related desynchronization in humans. Neurosci Lett 331: 29–32, 2002. doi:10.1016/s0304-3940(02)00825-x. 12359316

[3] Cassim F, Monaca C, Szurhaj W, Bourriez JL, Defebvre L, Derambure P, Guieu JD. Does post-movement β synchronization reflect an idling motor cortex? Neuroreport 12: 3859–3863, 2001. doi:10.1097/00001756-200112040-00051. 11726809

[4] Schnitzler A, Salenius S, Salmelin R, Jousmäki V, Hari R. Involvement of primary motor cortex in motor imagery: a neuromagnetic study. Neuroimage 6: 201–208, 1997. doi:10.1006/nimg.1997.0286. 9344824

[5] Illman M, Laaksonen K, Liljeström M, Jousmäki V, Piitulainen H, Forss N. Comparing MEG and EEG in detecting the ∼20-Hz rhythm modulation to tactile and proprioceptive stimulation. NeuroImage 215: 116804, 2020. doi:10.1016/j.neuroimage.2020.116804. 32276061

[6] Walker S, Monto S, Piirainen JM, Avela J, Tarkka IM, Parviainen TM, Piitulainen H. Older age increases the amplitude of muscle stretch-induced cortical β-band suppression but does not affect rebound strength. Front Aging Neurosci 12: 117, 2020. doi:10.3389/fnagi.2020.00117. 32508626 PMC7248310

[7] Salmelin R, Hari R. Spatiotemporal characteristics of sensorimotor neuromagnetic rhythms related to thumb movement. Neuroscience 60: 537–550, 1994. doi:10.1016/0306-4522(94)90263-1. 8072694

[8] Parkkonen E, Laaksonen K, Piitulainen H, Parkkonen L, Forss N. Modulation of the reverse similar 20-Hz motor-cortex rhythm to passive movement and tactile stimulation. Brain Behav 5: e00328, 2015. doi:10.1002/brb3.328. 25874163 PMC4396160

[9] Houdayer E, Labyt E, Cassim F, Bourriez JL, Derambure P. Relationship between event-related β synchronization and afferent inputs: analysis of finger movement and peripheral nerve stimulations. Clin Neurophysiol 117: 628–636, 2006. doi:10.1016/j.clinph.2005.12.001. 16427358

[10] Gaetz W, Cheyne D. Localization of sensorimotor cortical rhythms induced by tactile stimulation using spatially filtered MEG. Neuroimage 30: 899–908, 2006. doi:10.1016/j.neuroimage.2005.10.009. 16326116

[11] Laaksonen K, Kirveskari E, Mäkelä JP, Kaste M, Mustanoja S, Nummenmaa L, Tatlisumak T, Forss N. Effect of afferent input on motor cortex excitability during stroke recovery. Clin Neurophysiol 123: 2429–2436, 2012. doi:10.1016/j.clinph.2012.05.017. 22721651

[12] Pfurtscheller G. Functional brain imaging based on ERD/ERS. Vision Res 41: 1257–1260, 2001. doi:10.1016/S0042-6989(00)00235-2. 11322970

[13] Neuper C, Wortz M, Pfurtscheller G. ERD/ERS patterns reflecting sensorimotor activation and deactivation. Prog Brain Res 159: 211–222, 2006. doi:10.1016/S0079-6123(06)59014-4. 17071233

[14] Salmelin R, Hämäläinen M, Kajola M, Hari R. Functional segregation of movement-related rhythmic activity in the human brain. Neuroimage 2: 237–243, 1995. doi:10.1006/nimg.1995.1031. 9343608

[15] Pfurtscheller G, Stancak A Jr, Neuper C. Post-movement β synchronization. A correlate of an idling motor area? Electroencephalogr Clin Neurophysiol 98: 281–293, 1996. doi:10.1016/0013-4694(95)00258-8. 8641150

[16] Jurkiewicz MT, Gaetz WC, Bostan AC, Cheyne D. Post-movement β rebound is generated in motor cortex: evidence from neuromagnetic recordings. NeuroImage 32: 1281–1289, 2006. doi:10.1016/j.neuroimage.2006.06.005. 16863693

[17] Parkes LM, Bastiaansen MC, Norris DG. Combining EEG and fMRI to investigate the post-movement β rebound. NeuroImage 29: 685–696, 2006. doi:10.1016/j.neuroimage.2005.08.018. 16242346

[18] Muthukumaraswamy SD, Myers JF, Wilson SJ, Nutt DJ, Lingford-Hughes A, Singh KD, Hamandi K. The effects of elevated endogenous GABA levels on movement-related network oscillations. NeuroImage 66: 36–41, 2013. doi:10.1016/j.neuroimage.2012.10.054. 23110884

[19] Hall SD, Stanford IM, Yamawaki N, McAllister CJ, Ronnqvist KC, Woodhall GL, Furlong PL. The role of GABAergic modulation in motor function related neuronal network activity. NeuroImage 56: 1506–1510, 2011. doi:10.1016/j.neuroimage.2011.02.025. 21320607

[20] Gaetz W, Edgar JC, Wang DJ, Roberts TP. Relating MEG measured motor cortical oscillations to resting γ-aminobutyric acid (GABA) concentration. Neuroimage 55: 616–621, 2011. doi:10.1016/j.neuroimage.2010.12.077. 21215806 PMC3411117

[21] Yamawaki N, Stanford IM, Hall SD, Woodhall GL. Pharmacologically induced and stimulus evoked rhythmic neuronal oscillatory activity in the primary motor cortex in vitro. Neuroscience 151: 386–395, 2008. doi:10.1016/j.neuroscience.2007.10.021. 18063484

[22] Rossiter HE, Boudrias MH, Ward NS. Do movement-related β oscillations change after stroke? J Neurophysiol 112: 2053–2058, 2014. doi:10.1152/jn.00345.2014. 25080568 PMC4274928

[23] Espenhahn S, Rossiter HE, van Wijk BCM, Redman N, Rondina JM, Diedrichsen J, Ward NS. Sensorimotor cortex β oscillations reflect motor skill learning ability after stroke. Brain Commun 2: fcaa161, 2020. doi:10.1093/braincomms/fcaa161. 33215085 PMC7660041

[24] Liddle EB, Price D, Palaniyappan L, Brookes MJ, Robson SE, Hall EL, Morris PG, Liddle PF. Abnormal salience signaling in schizophrenia: the role of integrative β oscillations. Hum Brain Mapp 37: 1361–1374, 2016. doi:10.1002/hbm.23107. 26853904 PMC4790909

[25] Uhlhaas PJ, Linden DE, Singer W, Haenschel C, Lindner M, Maurer K, Rodriguez E. Dysfunctional long-range coordination of neural activity during Gestalt perception in schizophrenia. J Neurosci 26: 8168–8175, 2006. doi:10.1523/JNEUROSCI.2002-06.2006. 16885230 PMC6673788

[26] Degardin A, Houdayer E, Bourriez JL, Destee A, Defebvre L, Derambure P, Devos D. Deficient “sensory” β synchronization in Parkinson's disease. Clin Neurophysiol 120: 636–642, 2009. doi:10.1016/j.clinph.2009.01.001. 19208497

[27] Hall SD, Prokic EJ, McAllister CJ, Ronnqvist KC, Williams AC, Yamawaki N, Witton C, Woodhall GL, Stanford IM. GABA-mediated changes in inter-hemispheric β frequency activity in early-stage Parkinson's disease. Neuroscience 281: 68–76, 2014. doi:10.1016/j.neuroscience.2014.09.037. 25261686 PMC4222199

[28] Heida T, Poppe NR, de Vos CC, van Putten MJ, van Vugt JP. Event-related mu-rhythm desynchronization during movement observation is impaired in Parkinson's disease. Clin Neurophysiol 125: 1819–1825, 2014. doi:10.1016/j.clinph.2014.01.016. 24560131

[29] Pihko E, Nevalainen P, Vaalto S, Laaksonen K, Mäenpää H, Valanne L, Lauronen L. Reactivity of sensorimotor oscillations is altered in children with hemiplegic cerebral palsy: a magnetoencephalographic study. Hum Brain Mapp 35: 4105–4117, 2014. doi:10.1002/hbm.22462. 24522997 PMC6869593

[30] Hoffman RM, Wilson TW, Kurz MJ. Hand motor actions of children with cerebral palsy are associated with abnormal sensorimotor cortical oscillations. Neurorehabil Neural Repair 33: 1018–1028, 2019. doi:10.1177/1545968319883880. 31679451 PMC6920527

[31] Kurz MJ, Proskovec AL, Gehringer JE, Heinrichs-Graham E, Wilson TW. Children with cerebral palsy have altered oscillatory activity in the motor and visual cortices during a knee motor task. Neuroimage Clin 15: 298–305, 2017. doi:10.1016/j.nicl.2017.05.008. 28560154 PMC5440753

[32] Parkkonen E, Laaksonen K, Piitulainen H, Pekkola J, Parkkonen L, Tatlisumak T, Forss N. Strength of ∼20-Hz rebound and motor recovery after stroke. Neurorehabil Neural Repair 31: 475–486, 2017. doi:10.1177/1545968316688795. 28164736

[33] Tang CW, Hsiao FJ, Lee PL, Tsai YA, Hsu YF, Chen WT, Lin YY, Stagg CJ, Lee IH. β-Oscillations reflect recovery of the paretic upper limb in subacute stroke. Neurorehabil Neural Repair 34: 450–462, 2020. doi:10.1177/1545968320913502. 32321366 PMC7250642

[34] Espenhahn S, de Berker AO, van Wijk BCM, Rossiter HE, Ward NS. Movement-related β oscillations show high intra-individual reliability. NeuroImage 147: 175–185, 2017. doi:10.1016/j.neuroimage.2016.12.025. 27965146 PMC5315054

[35] Piitulainen H, Illman M, Laaksonen K, Jousmäki V, Forss N. Reproducibility of corticokinematic coherence. Neuroimage 179: 596–603, 2018. doi:10.1016/j.neuroimage.2018.06.078. 29964185

[36] Piitulainen H, Illman M, Jousmäki V, Bourguignon M. Feasibility and reproducibility of electroencephalography-based corticokinematic coherence. J Neurophysiol 124: 1959–1967, 2020. doi:10.1152/jn.00562.2020. 33112711

[37] Oldfield RC. The assessment and analysis of handedness: the Edinburgh inventory. Neuropsychologia 9: 97–113, 1971. doi:10.1016/0028-3932(71)90067-4. 5146491

[38] Piitulainen H, Bourguignon M, Hari R, Jousmäki V. MEG-compatible pneumatic stimulator to elicit passive finger and toe movements. Neuroimage 112: 310–317, 2015. doi:10.1016/j.neuroimage.2015.03.006. 25770989

[39] Taulu S, Simola J. Spatiotemporal signal space separation method for rejecting nearby interference in MEG measurements. Phys Med Biol 51: 1759–1768, 2006. doi:10.1088/0031-9155/51/7/008. 16552102

[40] Gramfort A, Luessi M, Larson E, Engemann DA, Strohmeier D, Brodbeck C, Goj R, Jas M, Brooks T, Parkkonen L, Hämäläinen M. MEG and EEG data analysis with MNE-Python. Front Neurosci 7: 267, 2013. doi:10.3389/fnins.2013.00267. 24431986 PMC3872725

[41] Uusitalo MA, Ilmoniemi RJ. Signal-space projection method for separating MEG or EEG into components. Med Biol Eng Comput 35: 135–140, 1997. doi:10.1007/BF02534144. 9136207

[42] David O, Kilner JM, Friston KJ. Mechanisms of evoked and induced responses in MEG/EEG. NeuroImage 31: 1580–1591, 2006. doi:10.1016/j.neuroimage.2006.02.034. 16632378

[43] Illman M, Laaksonen K, Liljeström M, Piitulainen H, Forss N. The effect of alertness and attention on the modulation of the β rhythm to tactile stimulation. Physiol Rep 9: e14818, 2021. doi:10.14814/phy2.14818. 34173721 PMC8234481

[44] Pfurtscheller G, Stancak A Jr, Edlinger G. On the existence of different types of central β rhythms below 30 Hz. Electroencephalogr Clin Neurophysiol 102: 316–325, 1997. doi:10.1016/S0013-4694(96)96612-2. 9146493

[45] Tallon-Baudry C, Bertrand O, Delpuech C, Permier J. Oscillatory γ-band (30-70 Hz) activity induced by a visual search task in humans. J Neurosci 17: 722–734, 1997. 15: doi:10.1523/JNEUROSCI.17-02-00722.1997. 8987794 PMC6573221

[46] Wilson TW, Heinrichs-Graham E, Becker KM. Circadian modulation of motor-related β oscillatory responses. Neuroimage 102: 531–539, 2014. doi:10.1016/j.neuroimage.2014.08.013. 25128712 PMC4252760

[47] Bardouille T, Bailey L, CamCAN Group. Evidence for age-related changes in sensorimotor neuromagnetic responses during cued button pressing in a large open-access dataset. Neuroimage 193: 25–34, 2019. doi:10.1016/j.neuroimage.2019.02.065. 30849530

[48] Fry A, Mullinger KJ, O'Neill GC, Barratt EL, Morris PG, Bauer M, Folland JP, Brookes MJ. Modulation of post-movement β rebound by contraction force and rate of force development. Hum Brain Mapp 37: 2493–2511, 2016. doi:10.1002/hbm.23189. 27061243 PMC4982082

[49] Hari R, Salmelin R. Human cortical oscillations: a neuromagnetic view through the skull. Trends Neurosci 20: 44–49, 1997. doi:10.1016/S0166-2236(96)10065-5. 9004419

[50] Cassim F, Szurhaj W, Sediri H, Devos D, Bourriez J, Poirot I, Derambure P, Defebvre L, Guieu J. Brief and sustained movements: differences in event-related (de)synchronization (ERD/ERS) patterns. Clin Neurophysiol 111: 2032–2039, 2000. doi:10.1016/s1388-2457(00)00455-7. 11068239

[51] Zhang X, Li H, Xie T, Liu Y, Chen J, Long J. Movement speed effects on β-band oscillations in sensorimotor cortex during voluntary activity. J Neurophysiol 124: 352–359, 2020. doi:10.1152/jn.00238.2020. 32579410

[52] Derambure P, Defebvre L, Bourriez JL, Cassim F, Guieu JD. Event-related desynchronization and synchronization. Reactivity of electrocortical rhythms in relation to the planning and execution of voluntary movement. Neurophysiol Clin 29: 53–70, 1999. doi:10.1016/s0987-7053(99)80041-0. 10093818

[53] Parkkonen E, Laaksonen K, Parkkonen L, Forss N. Recovery of the 20 Hz rebound to tactile and proprioceptive stimulation after stroke. Neural Plast 2018: 7395798, 2018. doi:10.1155/2018/7395798. 29681928 PMC5851173

[54] Hillebrand A, Barnes GR. A quantitative assessment of the sensitivity of whole-head MEG to activity in the adult human cortex. Neuroimage 16: 638–650, 2002. doi:10.1006/nimg.2002.1102. 12169249

[55] Nikulin VV, Brismar T. Long-range temporal correlations in α and β oscillations: effect of arousal level and test-retest reliability. Clin Neurophysiol 115: 1896–1908, 2004. doi:10.1016/j.clinph.2004.03.019. 15261868

[56] Martin-Buro MC, Garces P, Maestu F. Test-retest reliability of resting-state magnetoencephalography power in sensor and source space. Hum Brain Mapp 37: 1: 179–190, 2016. doi:10.1002/hbm.23027. 26467848 PMC6867588

[57] Heinrichs-Graham E, Wilson TW. Is an absolute level of cortical β suppression required for proper movement? Magnetoencephalographic evidence from healthy aging. Neuroimage 134: 514–521, 2016. doi:10.1016/j.neuroimage.2016.04.032. 27090351 PMC4912897

[58] Lemm S, Müller KR, Curio G. A generalized framework for quantifying the dynamics of EEG event-related desynchronization. PLoS Comput Biol 5: e1000453, 2009. doi:10.1371/journal.pcbi.1000453. 19662156 PMC2713829

[59] Laaksonen K, Helle L, Parkkonen L, Kirveskari E, Mäkelä JP, Mustanoja S, Tatlisumak T, Kaste M, Forss N. Alterations in spontaneous brain oscillations during stroke recovery. PLoS One 8: e61146, 2013. doi:10.1371/journal.pone.0061146. 23593414 PMC3623808

[60] Tecchio F, Zappasodi F, Pasqualetti P, Tombini M, Caulo M, Ercolani M, Rossini PM. Long-term effects of stroke on neuronal rest activity in rolandic cortical areas. J Neurosci Res 83: 1077–1087, 2006. doi:10.1002/jnr.20796. 16493681

[61] Gaetz W, Macdonald M, Cheyne D, Snead OC. Neuromagnetic imaging of movement-related cortical oscillations in children and adults: age predicts post-movement β rebound. Neuroimage 51: 792–807, 2010. doi:10.1016/j.neuroimage.2010.01.077. 20116434

[62] Xifra-Porxas A, Niso G, Larivière S, Kassinopoulos M, Baillet S, Mitsis GD, Boudrias MH. Older adults exhibit a more pronounced modulation of β oscillations when performing sustained and dynamic handgrips. Neuroimage 201: 116037, 2019. doi:10.1016/j.neuroimage.2019.116037. 31330245 PMC6765431

[63] Rossiter HE, Davis EM, Clark EV, Boudrias MH, Ward NS. Beta oscillations reflect changes in motor cortex inhibition in healthy ageing. Neuroimage 91: 360–365, 2014. doi:10.1016/j.neuroimage.2014.01.012. 24440529 PMC3988925

[64] Koelewijn L, Bompas A, Tales A, Brookes MJ, Muthukumaraswamy SD, Bayer A, Singh KD. Alzheimer's disease disrupts α and β-band resting-state oscillatory network connectivity. Clin Neurophysiol 128: 2347–2357, 2017. doi:10.1016/j.clinph.2017.04.018. 28571910 PMC5674981

[65] Toth M, Kiss A, Kosztolanyi P, Kondakor I. Diurnal alterations of brain electrical activity in healthy adults: a LORETA study. Brain Topogr 20: 63–76, 2007. doi:10.1007/s10548-007-0032-3. 17929159

[66] Hall SD, Barnes GR, Furlong PL, Seri S, Hillebrand A. Neuronal network pharmacodynamics of GABAergic modulation in the human cortex determined using pharmaco-magnetoencephalography. Hum Brain Mapp 31: 581–594, 2010. doi:10.1002/hbm.20889. 19937723 PMC3179593

[67] Jensen O, Goel P, Kopell N, Pohja M, Hari R, Ermentrout B. On the human sensorimotor-cortex β rhythm: sources and modeling. Neuroimage 26: 347–355, 2005. doi:10.1016/j.neuroimage.2005.02.008. 15907295

[68] Boksem MA, Meijman TF, Lorist MM. Effects of mental fatigue on attention: an ERP study. Brain Res Cogn Brain Res 25: 107–116, 2005. doi:10.1016/j.cogbrainres.2005.04.011. 15913965

[69] Kamiński J, Brzezicka A, Gola M, Wróbel A. Beta band oscillations engagement in human alertness process. Int J Psychophysiol 85: 125–128, 2012. doi:10.1016/j.ijpsycho.2011.11.006. 22155528

[70] Bardouille T, Picton TW, Ross B. Attention modulates β oscillations during prolonged tactile stimulation. Eur J Neurosci 31: 761–769, 2010. doi:10.1111/j.1460-9568.2010.07094.x. 20384818 PMC5005068

[71] Dockstader C, Cheyne D, Tannock R. Cortical dynamics of selective attention to somatosensory events. Neuroimage 49: 1777–1785, 2010. doi:10.1016/j.neuroimage.2009.09.035. 19781649

[72] Bauer M, Oostenveld R, Peeters M, Fries P. Tactile spatial attention enhances γ-band activity in somatosensory cortex and reduces low-frequency activity in parieto-occipital areas. J Neurosci 26: 490–501, 2006. doi:10.1523/JNEUROSCI.5228-04.2006. 16407546 PMC6674422

[73] Piitulainen H, Nurmi T, Hakonen M. Attention directed to proprioceptive stimulation alters its cortical processing in the primary sensorimotor cortex. Eur J Neurosci 54: 4269–4282, 2021. doi:10.1111/ejn.15251. 33955066

